# Supplementary material for: Optimal anti-amyloid-beta therapy for Alzheimer’s disease via a personalized mathematical model
Source: PLoS Comput Biol. 2022 Sep 2;18(9):e1010481. doi: 10.1371/journal.pcbi.1010481 (PMC9477429; doi:10.1371/journal.pcbi.1010481)
Supplement: Attachment — Submitted filename: PCOMPBIOL-D-22-00432-Report.pdf [file pcbi.1010481.s001.pdf]

Report on: Optimal Anti-amyloid-beta Therapy for Alzheimer's Disease via a Personalized Mathematical Model

by Wenrui Hao, Suzanne Lenhart, Jeffrey R. Petrella

In this paper the authors develop a modeling approach to provide a personalized optimal AD treatment plan for individual patients, using optimal control theory. They integrate personal longitudinal biomarker data from ADNI database into their model by fitting the personalized parameters (via optimal control theory). This modeling approach, though a simplification, is certainly very interesting and can concretely contribute, in my opinion, to the current research and clinical trials for AD.

I recommend publication of the paper after some minor concerns, that I list below, have been properly addressed by the authors.

Some minor concern:

(1) line 149, there is a typo, ref [2] should be ref [14];

(2) this is an old story between mathematicians and medical doctors: for me (a mathematician) the unknown in the equations are called "variables", while che coefficients are called "parameters". This is also the terminology used in the original paper by Hao and Friedman (2016). However medical doctors often call parameters the unknown (the variables in the equations). Unfortunately in this paper the word "parameter" is used for both sets, the "real" parameters but also the variables. Therefore I get sometimes confused on what comes from the the ADNI data and what are the outputs of the numerical solutions of the ODE. For example in the caption of Figure 3 it is not clear to me what are the parameter fitted... assuming the blu lines are the "variables" as functions of time, obtained as numerical solution of the ODE system...

(3) In Table 1 I don't understand what is the last block of data, i.e. the last 5 lines. I assume that the first two blocks (Initial conditions, Parameter values) are from the ADNI database, but the last one (Relative errors) what is it ? In comes from the solution of the ODE system? All the % symbols after error bars should be there?

(4) above line 145: the N in the formula for the relative error should be an n, referring to the number of elements of each group in Table 1;

(5) above line 157: there is a reason for this choice of parameters in the side-effect function ?
