# Supplementary material for: Optimal anti-amyloid-beta therapy for Alzheimer’s disease via a personalized mathematical model
Source: PLoS Comput Biol. 2022 Sep 2;18(9):e1010481. doi: 10.1371/journal.pcbi.1010481 (PMC9477429; doi:10.1371/journal.pcbi.1010481)
Supplement: Attachment — Submitted filename: AD_optimal_control_response.pdf [file pcbi.1010481.s002.pdf]

Response to reviewers' comments

We thank three reviewers for their valuable suggestions and comments. Accordingly, we have revised the manuscript to address all the comments (highlighted in red color) and also explain in detail below.

1 Reviewer 1

1. Even though AD clinical markers are used to determine individual parameters, some dataset only has three longitudinal datapoints in a short range of time. The starting time is chosen as $T_0 = 50$. Will the fitting results vary a lot with respect to the choice of the initial time? In the paragraph, it was mentioned that some longitudinal dataset is available for up to 10 years. Does the parameter fitting work well for these kinds of dataset?

Answer: AD patients develop symptoms around age 60. Thus the ADNI dataset collects the clinical data starting from age 54. Although ADNI dataset is available for up to 10 years, we use the ADNI-1 sub dataset in this paper for the optimal treatment study. We will explore other datasets in the future.

2. For Table 2, how can one tell whether they are for 78-week or 10-year treatments? Top versus bottom? As the numerical values ranges roughly from 10^{-7} to 29, the average may not be a good indicator. Maybe use median instead. Most readers will be benefited from understanding why the decline varies widely. In what situation, the decline will be small? In Table 3 and Table 4, some values are as small as 10^{-16} which is about the machine precision. This raises the concern of the accuracy of the numerical methods. Can authors address that? Are the results reliable?

Answer: We included a new column in Table 2 to distinguish 78-week and 10-year treatments. Following the reviewer's suggestion, we also change "average" to "median" in Tables 2-4. The widely varying range is a good justification of personalized medicine. Some patients benefit from the anti-Abeta treatment but some patients do not. The small numbers in Tables 2-4 mean that the patients do not have a response to the treatment. By considering the numerical accuracy, we set the number less than 10^{-7} as "NR" (stands for No Response) in the tables.

3. Here are some minor suggestions:

   Keywords: "Alzheimer's" instead of "alzheimer's" On page 9, one of the "%" in relative errors for A_β is misallocated.

   On page 10, caption in Fig.3: "with age 84.7" instead of "with 84.7".

   On page 11, line 172: "are in average 5.9%" instead of "are 5.9%". Is this for 78-week treatment? Also check the last sentence in this paragraph. Be specific in discussing the results that you have.

Answer: We have corrected typos.

2 Reviewer 2

1. (1) line 149, there is a typo, ref [2] should be ref [14];

Answer: Thanks for catching this typo.

(2) this is an old story between mathematicians and medical doctors: for me (a mathematician) the unknown in the equations are called "variables", while the coefficients are called "parameters". This is also the terminology used in the original paper by Hao and Friedman (2016). However medical doctors often call parameters the unknown (the variables in the equations). Unfortunately in this paper the word "parameter" is used for both sets, the "real" parameters but also the variables. Therefore I get sometimes confused on what comes from the the ADNI data and what are the outputs of the numerical solutions of the ODE. For example in the caption of Figure 3 it is not clear to me what are the parameter fitted... assuming the blue lines

are the “variables” as functions of time, obtained as numerical solution of the ODE system...

Answer: The present manuscript is different from the reference by Hao and Friedman (2016). In that paper, all the parameters were estimated by the experiments in literature. In another words, they set all the parameters as coefficients in the model before doing any simulations. However, in the present paper, we fit the parameter values by using biomarker data in ADNI dataset. In this case, the parameters are not given but computed via solving the optimization problem (8). Thus the parameters are also “unknown.” But once we solved the optimization problem, the parameter values are fixed as coefficients of the ODE for the optimal control study. In Figure 3, we show the numerical solutions of best-fitted parameter values (blue lines).

(3) In Table 1 I don’t understand what is the last block of data, i.e. the last 5 lines. I assume that the first two blocks (Initial conditions, Parameter values) are from the ADNI database, but the last one (Relative errors) what is it ? In comes from the solution of the ODE system? All the % symbols after error bars should be there?

Answer: The first two blocks, Initial conditions and Parameter values, are fitted by using ADNI dataset. The relative errors are used to evaluate the fit quality and are defined by

$$\sqrt{\frac{1}{n}\sum_{i=1}^{n}\frac{(x(t_i)-\tilde{x}_i)^2}{\tilde{x}_i^2}}$$

where $x(t_i)$ is the model value of the biomarker while $\tilde{x}_i$ is the clinical measurement at age t_i. We explained this in **Personalized parameters 154** of **Results** section.

(4) above line 145: the N in the formula for the relative error should be an n, referring to the number of elements of each group in Table 1;

Answer: We have changed to n in the revised manuscript.

(5) above line 157: there is a reason for this choice of parameters in the side-effect function ?

Answer: We explained in more detail the choice of parameters for the side-effect function in the revised manuscript.

3 Reviewer 3

1. To fully judge the importance and level of reliability of the model, it would be important to have information about the optimality of single personalized trials. Do the authors have any indications for the reliability of the optimization of the trial for single patients? If not, do they have any argument to convince the reader of such reliability, or, at least, can they indicate how to handle this issue in the future? This would make the paper more valuable.

Answer: We validate the model by using the ADNI dataset for each single patient by calibrating the parameters and the initial conditions. We have shown the relative errors for each biomarker to evaluate the good fit quality in Table 1. However, in order to validate the optimization of the trial, we need to have access to the clinical trail data which is unfortunately not available publicly. We put this as one of our future directions in the conclusion part of the revised manuscript.
