# Supplementary material for: Optimal anti-amyloid-beta therapy for Alzheimer’s disease via a personalized mathematical model
Source: PLoS Comput Biol. 2022 Sep 2;18(9):e1010481. doi: 10.1371/journal.pcbi.1010481 (PMC9477429; doi:10.1371/journal.pcbi.1010481)
Supplement: Attachment — Submitted filename: Plos_response.pdf [file pcbi.1010481.s003.pdf]

Response to reviewer 1' comments

According to reviewer 1's minor suggestions, we have revised the manuscript and highlighted the changes in red color.

1 Reviewer 1

1. On page 2, line 39: remove the space after taupathology.

   On page 8, line 148: add a space after (6).

Answer: We have made these minor changes accordingly.

2. It seems that $\lambda_{\tau A_\beta}$ and λ_τ refer to the same parameter. It is better to make it consistent across the manuscript.

Answer: Thanks for catching this. We use λ_τ in the revised manuscript.
